# Incidence, Severity, and Outcomes of Acute Kidney Injury in Octogenarians following Heart Valve Replacement Surgery

**DOI:** 10.1155/2015/237951

**Published:** 2015-05-24

**Authors:** Michael A. Mao, Charat Thongprayoon, YiFan Wu, Vickram Tejwani, Myriam Vela-Ortiz, Joseph Dearani, Qi Qian

**Affiliations:** ^1^Division of Nephrology and Hypertension, Department of Medicine, Mayo Clinic College of Medicine, Rochester, MN 55905, USA; ^2^Division of Anesthesiology, Mayo Clinic College of Medicine, Rochester, MN 55905, USA; ^3^Department of Surgery, Mayo Clinic College of Medicine, Rochester, MN 55905, USA

## Abstract

*Background*. The study investigates the occurrence, severity, and outcomes of acute kidney injury (AKI) in octogenarians following heart valve surgery.* Methods*. All patients, age >80 years, not on dialysis and without kidney transplant, undergoing heart valve replacement at Mayo Clinic, Rochester, in the years 2002-2003 were enrolled. AKI was diagnosed based on AKIN criteria.* Results*. 209 octogenarians (88.0% aortic valve, 6.2% mitral valve, 1.0% tricuspid valve, and 4.8% multivalve) with (58.4%) and without CABG were studied. 34 (16.3%) had preexisting CKD. After surgery, 98 (46.8%) developed AKI. 76.5% of the AKI were in Stage 1, 9.2% in Stage 2, and 14.3% in Stage 3. 76.5% CKD patients developed AKI. Length of hospital stay was longer for AKI patients. More AKI patients were discharged to care facilities. Patient survival at 30 days and 1 year for AKI versus non-AKI was 88.8 versus 98.7%, *p* = 0.003, and 76.5 versus 88.3%, *p* = 0.025, respectively. With follow-up of 3.94 ± 0.28 years, Kaplan-Meier analysis showed a reduced survival for AKI octogenarians. Preexisting CKD and large volume intraoperative fluid administration were independent AKI predictors.* Conclusions*. Nearly half of the octogenarians developed AKI after valve replacement surgery. AKI was associated with significant functional impairment and reduced survival.

## 1. Introduction

Population is aging in the United States and nearly worldwide [1]. The US Census Bureau projects that the very elderly population (age ≥ 80) will grow from 5.8 million (1.8% of the population) in 2012 to 13 million (3.2%) by 2050 [2]. Heart valves are well known to degenerate with aging, affecting up to 13.2% patients age ≥75 years [3]. With limited nonsurgical treatment options for valvular heart diseases and significant clinical morbidity and mortality, it is foreseeable that valve replacement operations will be performed for more elderly in the future [3–7]. With limited organ function reserve, increasing comorbidities, and reduced adaptive capacity, the elderly are at high risk for postoperative complications such as AKI [8]. Aging alone is a significant risk factor for AKI [9], and the severity of AKI is proportional to the poor outcomes including morbidity and mortality [10]. Data on the incidence, severity, and outcomes of AKI in octogenarians undergoing heart valve surgery is, however, scarce [5].

The aim of this study is to determine AKI occurrence, severity, and outcomes in a cohort of octogenarians undergoing valve replacement surgery. We also explore potential predictors for the development of AKI in this cohort.

## 2. Patients and Methods

### 2.1. Data Collection and AKI Definitions

The Institutional Review Board approved the study. Between 2002 and 2003, 210 octogenarians with symptomatic cardiac valve disease underwent valve replacement surgery who were not on hemodialysis and without kidney transplant. The Charlson Comorbidity Index at the time of admission was collected for each patient [11]. Survival was censored based on death dates in the institutional records and publically accessible Social Security Death Index http://www.genealogybank.com/gbnk/ssdi/.

Preexisting chronic kidney disease (CKD) was defined based on primary physician's documentation within six months of the surgery. Baseline serum creatinine (s.Cr, in mg/dL) was defined as the s.Cr measurement within three months of the index admission. Estimated GFR was calculated based on the Chronic Kidney Disease Epidemiology Collaboration (CKD-EPI) [12]. The patients were grouped based on their eGFR. One patient who expired intraoperatively was excluded from analysis.

Acute kidney injury (AKI) was defined as an abrupt (within 48 hours) increase in s.Cr ≥0.3 mg/dL or s.Cr increase by 50% from baseline within seven days postoperatively. The AKIN urinary criteria were omitted due to incomplete data on urine output [13]. The severity of AKI was determined using s.Cr definitions from the AKIN criteria [13]. The AKIN criteria classify AKI into the following: Stage 1, s.Cr elevation ≥ 1.5 times the baseline s.Cr; Stage 2, ≥2 times the baseline s.Cr; Stage 3, ≥3 times the baseline s.Cr, s.Cr ≥ 4.0 mg/dL or complete loss of kidney function requiring renal replacement therapy.

Net fluid balance equaled to fluid input minus fluid output (in liters).

### 2.2. Statistical Analysis

Statistical analysis was performed using JMP version 10.0.0. For categorical variables, Fisher's exact test and Pearson's chi-squared test were utilized. For continuous variables, Student's *t*-test was applied for comparison between the AKI and non-AKI cohorts. The results are reported as percentage frequencies and means ± standard deviation (SD). *p* value of <0.05 (two-tailed) was considered significant. Stepwise backward logistic regressions were performed to derive the final multivariate model taking into consideration colinearity, interaction, and number of patients who expressed the outcome of interest. Cox proportional hazard regression modeling was used to compare survival in the two cohorts adjusted for age and comorbidity. Kaplan-Meier analysis was used to compare the long-term survival.

## 3. Results

### 3.1. Patient Characteristics

The 209 octogenarians represented 16% of the total patients undergoing valve replacement surgery at Mayo Clinic Rochester in 2002 and 2003. 184 (88.0%) of the 209 underwent aortic valve replacement (AVR), 13 (6.2%) mitral valve replacement (MVR), 2 (1.0%) tricuspid valve replacement (TVR), and 10 (4.8%) combined valve replacement (6 AV-MV, 1 AV-TV, 2 MV-TV, and 1 PV-TV). All but one patient had bioprosthetic replacement valves. Preoperative characteristics of the cohort showed a median age of 84.1 years and a mean Charlson comorbidity score [11] of 2.63 ± 1.76 (Table 1). 127 (60.8%) patients had preexisting hypertension and 35 (16.7%) patients had a prior myocardial infarction. Thirty-four (16.3%) patients had a diagnosis of CKD with a mean s.Cr of 1.7 ± 0.31 mg/dL corresponding to an eGFR of 34.3 ± 10.0 mL/min/BSA, whereas 175 non-CKD patients had s.Cr of 1.15 ± 0.20 mg/dL, eGFR of 52.8 ± 11.0 mL/min/BSA, *p* < 0.0001 (Table 2).

### 3.2. AKI Occurrence and Severity

Ninety-eight of 209 patients (46.8%) developed postoperative AKI, of which 13 (6.2%) required renal replacement therapy (7 from the CKD group and 6 from the non-CKD group). The majority of AKIs were within AKIN Stage 1 (risk). Patients with preexisting CKD were at higher risk for postoperative AKI, 26 (76.5%) versus 72 (41.1%) in non-CKD, *p* = 0.0002. AKI in CKD patients were more severe, 26.9% versus 9.7% in Stage 3 (Table 2).

Prior studies show that, among octogenarians undergoing AVR, age ≥ 84 years had the highest odds ratio for 6-month mortality [14]. We examined AKI occurrence in octogenarians age <84 and ≥84. The eGFR was different, 52.5 mL/min/BSA in those age <84 (*N* = 101) and 47.2 mL/min/BSA in age ≥84 (*N* = 108), *p* = 0.003. However, the AKI occurrence between the two age groups did not differ, 44.4 versus 49.5%, *p* > 0.05. The proportion of AKI patients in Stages 1, 2, and 3 was also not different in the two age groups. The 6-month mortality rate was likewise comparable, 12.9 versus 12.0%, *p* > 0.05.

We also compared those with and without concomitant coronary artery bypass grafting (CABG), as previous studies had shown conflicting results on the effect of concomitant CABG on mortality [5, 7, 14]. In our cohort, CABG did not increase postoperative AKI. One-year mortality rate in the two groups was comparable, 13.9% with CABG versus 21.8% without CABG, *p* = 0.14. The number of bypassed vessels did not affect the 30-day, 6-month, or 1-year mortality (data not shown).

### 3.3. Characteristics of AKI versus Non-AKI Patients

Patient characteristics in AKI and non-AKI were compared. AKI patients tended to have preexisting CKD, prior myocardial infarction, higher Charlson Comorbidity Index, and lower preoperative hemoglobin (Table 3). Intraoperative factors associated with AKI included longer surgical duration, intra-aortic balloon pump use, lower intraoperative nadir hemoglobin, and higher volume of blood transfusion and fluids/saline infusion (Table 3). Additionally, AKI patients were possibly more likely to have a higher BMI and atherosclerotic vascular diseases, although *p* value did not reach statistical significance.

### 3.4. Predictors for AKI

Potential AKI predictors in this cohort of octogenarians were explored. Univariate analysis showed prior MI, CKD, higher Charlson Comorbidity Index, lower preoperative hemoglobin, longer surgical duration, blood transfusion, and intraoperative fluid and saline administration as potential risk factors. Two models of multivariate stepwise backward logistic regression analysis were built. Preexisting CKD (OR 4.87–5.38, 95% CI 2.12–13.6, *p* < 0.001), intraoperative fluid administration (OR 1.10, CI 1.01–1.21, *p* = 0.03) and intraoperative saline infusion (OR 1.52, CI 1.05–2.24, *p* = 0.02) were shown as independent predictors for AKI (Tables 4(a) and 4(b)).

### 3.5. Length of Hospital Stay and Disposition

**Figure 1:**
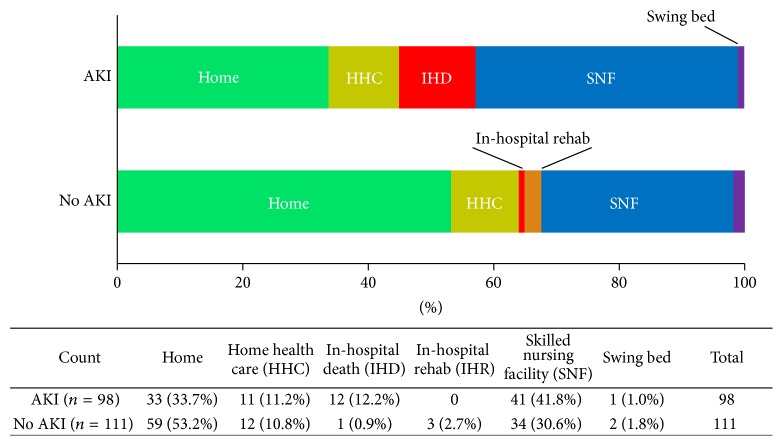
Patient disposition.

Length of hospital stay (LOS) was longer for AKI patients 14.0 ± 11.7 days versus 9.2 ± 4.2 days for non-AKI patients, *p* < 0.0001. Patient disposition was different in octogenarians with and without AKI (Figure 1, *p* = 0.001). More AKI patients discharged to skilled nursing facilities (41.8% AKI versus 30.6% non-AKI) and fewer to home (33.7% AKI versus 53.2% non-AKI), reflecting a higher level of functional impairment in patients with AKI, which is known to be associated with a high long-term mortality rate [15]. The disposition to a swing bed, another hospital adjacent to patient's home, was not different between the AKI and non-AKI groups.

### 3.6. Renal and Patient Outcomes

**Figure 2:**
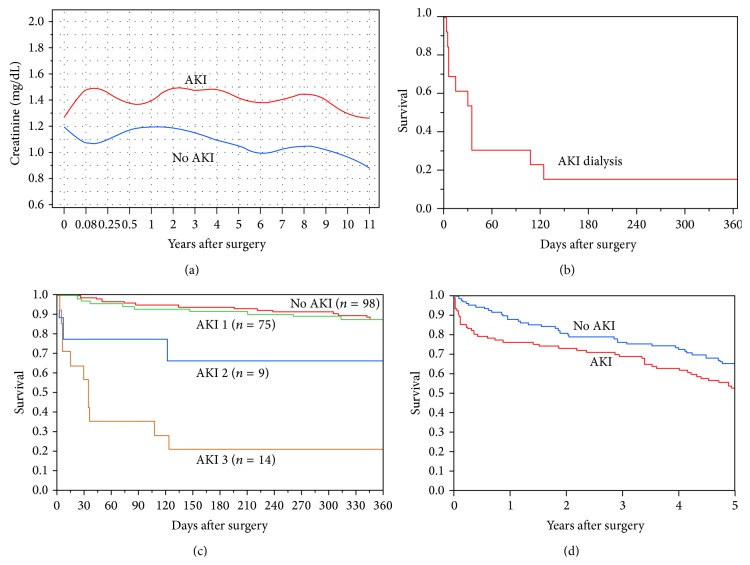
Patient survival. (a) Long-term s.Cr in AKI and non-AKI octogenarians. (b) Kaplan-Meier 1-year survival in octogenarians with AKI requiring dialysis (*n* = 13). (c) Kaplan-Meier 1-year survival by AKI Stages versus non-AKI, unadjusted log-rank *p* < 0.0001. (d) Kaplan-Meier survival curves, AKI versus non-AKI patients, log-rank *p* = 0.016. The adjusted^a^ HR for AKI 2.02; 95% CI 1.01–4.04, *p* = 0.04.

Among surviving AKI patients, s.Cr at the time of hospital discharge was higher in AKI patients, 1.46 ± 0.56 mg/dL versus 1.09 ± 0.25 mg/dL in non-AKI patients, *p* < 0.0001. Long-term follow-up for s.Cr values after hospitalization was limited by our hospital being a large referral center. Many patients received their nonsurgical care at local care facilities geographically removed from Mayo Clinic. For those who continued regular nonsurgical follow-up with us, a persistent separation of s.Cr values between the AKI and non-AKI groups was observed (Figure 2(a)).

Early postoperative mortality rates were dramatically different in AKI versus non-AKI octogenarians. In-hospital mortality in AKI patients was >10-fold higher than the mortality in non-AKI octogenarians, 12.2% versus 0.9%, *p* = 0.0003. 30-day postsurgery mortality for AKI group was 10.2% versus 0.9% in non-AKI groups, *p* = 0.003. For the 13 AKI patients who required dialysis, 46% died within 30 days after surgery, 84.6% died within 1 year, and 92.3% died within 5 years (Figure 2(b)). Only two of the dialysis patients were able to come off dialysis at the time of discharge.

Late postoperative mortality rates were different between AKI and non-AKI octogenarians. AKI patients showed a 1-year mortality of 23.5% versus 11.7% in non-AKI patients, *p* = 0.02.  Figure 2(c) shows 1-year survival in non-AKI and AKI (Stages 1–3) octogenarians. Kaplan-Meier analysis of survival in non-AKI and AKI octogenarians with a mean follow-up of 3.94 ± 4.04 years demonstrates a higher mortality rate in postoperative AKI octogenarians than those without AKI, *p* = 0.02 (Figure 2(d)).

Unadjusted Cox proportional hazard model showed that AKI Stage 3 was a significant contributor to 1-year patient mortality and AKI Stage 2 was nearly significant (Table 5(a)). Cox proportional hazard model adjusted for age and Charlson comorbidity score showed that AKI Stage 3 independently contributed to the 1-year mortality, HR 13.6, 95% CI: 5.4–33.9, *p* < 0.0001 (Table 5(b)).

## 4. Comment

In this study of octogenarians undergoing heart valve replacement surgery, we show that the incidence of postoperative AKI was nearly 50%. Moreover, AKI occurrence is associated with a prolonged hospital stay, increased need for higher level of care following discharge, persistently reduced kidney function, and reduced short-term and long-term patient survival.

Demand for heart valvular surgery in octogenarians is expected to increase as the population ages [3–7, 16]. Studies have shown that valve replacement surgeries have acceptable mortality rates in the very elderly [6, 16]. At Mayo Clinic Rochester, over 16% of valve replacement surgeries in 2002 and 2003 were performed for octogenarians. Although less invasive transcatheter aortic valve implantation (TAVI) has recently been introduced into practice, there has been wide center-to-center variation in the rates of postprocedural complications (AKI, stroke, and mortality), 4.7 to over 20% [17, 18], and the procedure is limited to the aortic valve replacement. Conventional valve surgery remains the standard of care.

Older age and preexisting CKD are major risk factors for AKI [19, 20]. AKI in hospitalized patients tends to progress to end-stage renal failure, especially for the elderly [21], and raises mortality. AKI is known to occur after cardiac surgery and is correlated with mortality [22]. Studies in octogenarians undergoing heart valve replacement have been limited. Available studies examine primarily postoperative mortality; when renal impairments are mentioned, no specific definitions, such as AKIN/RIFLE criteria, were applied [6, 7, 23–25]. The 30-day mortality rates in most studies range from 3.4 to 8.5% after aortic valve replacement [6, 7, 14, 16, 23–28] and 18.2 to 18.5% after mitral valve replacement [5]. One-year mortality ranges from 7.1 to 35%. [6, 7, 14, 25, 28]. The current study, involving all valve replacement surgeries in 209 octogenarians, shows a 30-day mortality of ~5% and 1-year mortality of 17%, roughly in line with previous study results. New to the existing literature is that deaths in this cohort of octogenarians occurred almost exclusively in those with AKI (Table 5(a)). Notably, a small preoperative s.Cr difference of ~0.5 mg/dL between non-CKD and CKD patients reflected an eGFR difference of ~20 mL/min/BSA (from 52.8 to 34.3 mL/min/BSA) and nearly a doubling of AKI occurrence. Moreover, longer follow-up allowed for long-term outcome assessment, showing a persistently poor kidney function and higher mortality among AKI octogenarians.

Predictors for AKI by univariate analysis in this cohort of octogenarians were a higher Charlson Comorbidity Index, preexisting CKD, prior myocardial infarction, preoperative anemia, surgical duration, intraoperative fluid administration, low intraoperative hemoglobin, and blood transfusion. Stepwise multivariate analysis adjusting for relevant univariate risk factors showed that CHF, CKD, total intraoperative fluids, and saline infusion are independent predictors for postoperative AKI in this cohort of octogenarians (Tables 4(a) and 4(b)). Excessive intravenous fluids, especially 0.9% saline, have been associated with the development of postoperative complications and AKI [29–32]. Volume overload in the setting of AKI has been shown to be associated with poor clinical outcomes including mortality [33].

Cox proportional hazard regression was performed to explore survival differences between AKI and non-AKI octogenarians adjusted for age and comorbidity (Table 5). Mortality rates in AKI octogenarians were ~10%, 25%, and 50% in 30 days, 1 year, and 5 years, respectively. Although difficult to make a direct comparison, our results are not inferior to the mortality rates in previous studies inclusive of adult AKI patients of all ages showing in-hospital mortality rates of ~20–50% [10, 34, 35]. Among 13 dialysis octogenarians in the current study, 46% died at 30 days after operation. Despite a high mortality rate, it is not inferior to the results generated in nonelderly adult AKI-dialysis patients of 55% [36].

In the current study, concomitant CABG or number of coronary vessels bypassed did not increase the rate of AKI (Table 2) or mortality, consistent with previous studies showing no mortality impact in concomitant CABG to valve surgery [5, 7]. There were, however, also studies showing an increase or decrease in mortality in valve surgery with CABG [14, 23, 24]. Given the limited patient number, our results should be viewed with caution. Further study with larger patient number is necessary.

Florath et al. [14] reported mortality rates of 8.4% at 30 days, 15.2% at 6 months, and 26% at 1 year in octogenarians undergoing AVR. An age >84 years was related to poor survival. We did not find age >84 to have been associated with higher AKI or mortality. This discrepancy could have been due to a small difference in eGFR and similar Charlson Index in our cohorts. Chiappini et al. showed [26] that the predictors for mortality in octogenarians following AVR are poor preoperative EF and heart failure. Our data did not show these being the significant risks for AKI or mortality, although our study patients might be different from theirs. No studies, however, have explored in detail the relationship between post-valve-surgery AKI and patient's outcomes including disposition, long-term kidney function, and patient survival in octogenarians, as shown in the current study.

Approximately two-thirds of AKI progress to a severe stage [37], which is associated with a further increase in mortality. Nephrology involvement in their care can potentially mitigate progression of AKI and its sequelae. Our results support the early involvement of nephrology in the care of octogenarians with AKI, especially for those with reduced preoperative eGFR and those with a small increase in postsurgery s.Cr (≥0.3 mg/dL). In practice, nephrology referral for octogenarian AKI patients has been low at ~20% [38]. That said, confirmatory evidence of the beneficial effects of nephrology involvement should be further studied.

Several limitations in this study should be considered. First, the nature of the observational cohort study limited consistency in the timing of laboratory testing for creatinine and hemoglobin values, which could influence their values. However, surgical patients are unique in that we knew precisely the time of insult (surgery). Furthermore, a single center study prevented large variation in practice and routine blood draws were relatively fixed in time of day. Second, our data were collected in 2002 and 2003, which may not precisely reflect present-day outcomes. However, there has not been a major practice change in valve replacement surgery, and data over an uninterrupted period allowed longitudinal follow-up of patients' outcomes. Third, the majority of our patients are Caucasian, with only one black patient and a few patients of other races, which limits the application of our results to other races. Fourth, AKI patients received more intravenous fluids, which could dilute s.Cr and underestimate AKI severity. However, even with this limitation, we still found significant poor clinical outcomes associated with AKI. Fifth, data on the administration of hydroxyethyl starch (HES) and aprotinin, known to be associated with AKI [39, 40], were not analyzed because these agents were used occasionally and unlikely to have affected the results. Lastly, the association between AKI and poor clinical outcomes could potentially be affected by the preexisting CKD. The strong effect of CKD may hinder the determination of true impact of AKI on patients' outcomes. Nonetheless, severe AKI stood out as an independent risk factor for mortality in our cohort. We believe that even mild-to-moderate degrees of AKI could potentially exert negative impact on patient survival, although the signal may have been overwhelmed by the extraordinarily strong effect of preexisting CKD. Taken together, CKD is a strong reason for increased mortality after AKI, but AKI independently contributed to the poor patient outcomes.

Overall, our data show that octogenarians are able to tolerate heart valve replacement surgery. However, the AKI occurrence rate is substantial and AKI impacts patients' mortality and morbidity. Better patient selection, preoperative preparation, and avoidance of operative risk factors can minimize the AKI risk and adverse consequences.

## Figures and Tables

**Table 1:** Preoperative characteristics of octogenarians.

Baseline characteristics	*N* = 209
Age (year) at surgery, median [range]	84.1 [80.1–93.4]
Males (%)	115 (55.0)
Race (%)	Caucasian 175 (83.7)
	Other: 32 (15.3)
	Black: 1 (0.48)
BMI, mean ± SD	27.7 ± 5.3
HTN (%)	127 (60.8)
Diabetes (%)	40 (19.1)
CKD (%)	34 (16.3)
CHF (%)	167 (79.9)
Prior MI (%)	35 (16.7)
Hyperlipidemia on lipid-lowering agents (%)	104 (49.8)
Charlson Index, mean ± SD	2.63 ± 1.76
Baseline Cr, mean ± SD	1.24 ± 0.30
eGFR, mean ± SD	49.79 ± 12.79
Pre-op EF %, mean ± SD	54.6 ± 14.9

**Table 2:** AKI occurrence and severity in CKD and non-CKD patients.

Baseline CKD status	All *n* = 209	CKD *n* = 34	Non-CKD *n* = 175	*p* value
Presurgery s.Cr, mg/dL	1.24 (0.30)	1.70 ± 0.31	1.15 ± 0.20	<0.0001
Presurgery eGFR	49.8 (12.8)	34.3 ± 10.0	52.8 ± 11.0	<0.0001
Calculated CKD stage^∗^				
CKD Stage 1	0	0	0	
CKD Stage 2	45 (21.5)	1 (2.9)	44 (25.1)	0.004
CKD Stage 3a	93 (44.5)	4 (11.8)	89 (50.9)	<0.0001
CKD Stage 3b	57 (27.3)	17 (50.0)	40 (22.9)	0.0011
CKD Stage 4	14 (6.7)	12 (35.3)	2 (1.1)	<0.0001
CKD Stage 5	0	0	0	
Total AKI number (%)	98 (46.8)	26 (76.5)	72 (41.1)	0.0002
Stage 1	75 (76.5)	17 (65.4)	58 (80.6)	0.0001
Stage 2	9 (9.2)	2 (7.7)	7 (9.7)	
Stage 3	14 (14.3)	7 (26.9)^a^	7 (9.7)^b^	

^a^7 of 7 in CKD and ^b^6 of 7 in non-CKD required dialysis.

^∗^Stratified by eGFR instead of physician's diagnosis of CKD.

**Table 3:** Preoperative characteristics and hospital course in AKI and non-AKI octogenarians.

Preoperative characteristics	No AKI, mean ± SD, *n* = 111	AKI, mean ± SD, *n* = 98	*p* value
Age, median [range]	84.1 ± 2.8 [80.1–93.4]	83.9 ± 2.8 [80.1–92.0]	0.49
Sex, male (%)	57 (51.3)	58 (59.2)	0.26
BMI, kg/m^2^	27.1 ± 4.9	28.4 ± 5.7	0.09
HTN (%)	72 (64.9)	55 (56.1)	0.20
Diabetes (%)	18 (16.2)	22 (22.4)	0.25
Hyperlipidemia on lipid-lowering agents (%)	62 (55.9)	42 (42.9)	0.06
Chronic pulmonary disease (%)	19 (17.1)	14 (14.3)	0.58
Preexisting CKD (%)	8 (7.2)	26 (26.5)	<0.0001
Prior myocardial infarction (%)	12 (10.8)	23 (23.5)	0.01
CAD (%)	78 (70.3)	65 (66.3)	0.54
Peripheral vascular, cerebrovascular, or carotid artery disease (%)	29 (26.1)	36 (36.7)	0.10
CHF (%)	94 (84.7)	73 (74.5)	0.07
Charlson Comorbidity Index	2.39	2.91	0.03
Pre-op EF, %	53.7 ± 15.5	55.7 ± 14.3	0.34
Pre-op hemoglobin, g/dL	12.8 ± 1.6	11.9 ± 1.6	0.03

Hospital course			

Surgical duration, min	331.0 ± 82.8	369.1 ± 108.5	0.005
CABG, yes or no (%)	64 (57.7)	58 (59.2)	0.82
IABP (%)	2 (1.80)	9 (9.18)	0.02
Cross clamp time, min	69.7 ± 30.1	69.0 ± 32.3	0.85
CPB, min	96.7 ± 39.3	101.3 ± 43.3	0.42
Net intraoperative fluid balance, L	6.87 ± 2.57	8.12 ± 4.28	0.01
Total intraoperative saline, L	0.90 ± 0.72	1.16 ± 0.95	0.02
Intraoperative hemoglobin, g/dL^a^	8.28 ± 1.04	7.92 ± 1.04	0.01
RBC on operative day, L	0.74 ± 0.63	1.06 ± 1.03	0.008
RBC during hospitalization, L	0.92 ± 0.86	1.54 ± 1.65	0.0007
Length of hospital stay, day	9.2 ± 4.2 (*n* = 110)	14.0 ± 11.7 (*n* = 86)	<0.0001
Discharge creatinine, mg/dL	1.09 ± 0.25	1.46 ± 0.56	<0.0001

^a^The lowest hemoglobin during the operation.

**Table:** (a) Model number 1

Variables	OR (95% CI)	*p* value
CHF	0.43 (0.21–0.88)	0.02
CKD	4.87 (2.12–12.3)	<0.001
Intraoperative fluid volume	1.10 (1.01–1.21)	0.03

**Table:** (b) Model number 2

Variables	OR (95% CI)	*p* value
CHF	0.43 (0.21–0.87)	0.02
CKD	5.38 (2.34–13.6)	<0.001
Intraoperative saline volume	1.52 (1.05–2.24)	0.02

**Table:** (a)

AKI stage	HR (95% CI)	*p* value
No AKI	1	Reference
Stage 1	1.04 (0.4–2.4)	0.92
Stage 2	3.7 (0.8–11.4)	0.08
Stage 3	15.9 (6.9–36.3)	<0.0001

Unadjusted.

**Table:** (b)

AKI stage	HR (95% CI)	*p* value
No AKI	1	Reference
Stage 1	1.0 (0.4–2.4)	0.95
Stage 2	3.1 (0.7–10.3)	0.13
Stage 3	13.6 (5.4–33.9)	<0.0001

Adjusted for age and Charlson comorbidity score.
